# Machine learning model based on enhanced CT radiomics for the preoperative prediction of lymphovascular invasion in esophageal squamous cell carcinoma

**DOI:** 10.3389/fonc.2024.1308317

**Published:** 2024-02-23

**Authors:** Yating Wang, Genji Bai, Min Huang, Wei Chen

**Affiliations:** Department of Radiology, The Affiliated Huaian No.1 People’s Hospital of Nanjing Medical University, Huaian, Jiangsu, China

**Keywords:** radiomics, machine learning, ESCC, lymphovascular invasion, computed tomography

## Abstract

**Objective:**

To evaluate the value of a machine learning model using enhanced CT radiomics features in the prediction of lymphovascular invasion (LVI) of esophageal squamous cell carcinoma (ESCC) before treatment.

**Methods:**

We reviewed and analyzed the enhanced CT images of 258 ESCC patients from June 2017 to December 2019. We randomly assigned the patients in a ratio of 7:3 to a training set (182 cases) and a validation (76 cases) set. Clinical risk factors and CT image characteristics were recorded, and multifactor logistic regression was used to screen independent risk factors of LVI of ESCC patients. We extracted the CT radiomics features using the FAE software and screened radiomics features using maximum relevance and minimum redundancy (MRMR) and least absolute shrinkage and selection operator (LASSO) algorithms, and finally, the radiomics labels of each patient were established. Five machine learning algorithms, namely, support vector machine (SVM), K-nearest neighbor (KNN), logistic regression (LR), Gauss naive Bayes (GNB), and multilayer perceptron (MLP), were used to construct the model of radiomics labels, and its clinical features were screened. The predictive efficacy of the machine learning model for LVI of ESCC was evaluated using the receiver operating characteristic (ROC) curve.

**Results:**

Tumor thickness [OR = 1.189, 95% confidence interval (CI) 1.060–1.351, *P* = 0.005], tumor-to-normal wall enhancement ratio (TNR) (OR = 2.966, 95% CI 1.174–7.894, *P* = 0.024), and clinical N stage (OR = 5.828, 95% CI 1.752–20.811, *P* = 0.005) were determined as independent risk factors of LVI. We extracted 1,316 features from preoperative enhanced CT images and selected 14 radiomics features using MRMR and LASSO to construct the radiomics labels. In the test set, SVM, KNN, LR, and GNB showed high predictive performance, while the MLP model had poor performance. In the training set, the area under the curve (AUC) values were 0.945 and 0.905 in the KNN and SVM models, but these decreased to 0.866 and 0.867 in the validation set, indicating significant overfitting. The GNB and LR models had AUC values of 0.905 and 0.911 in the training set and 0.900 and 0.893 in the validation set, with stable performance and good fitting and predictive ability. The MLP model had AUC values of 0.658 and 0.674 in the training and validation sets, indicating poor performance. A multiscale combined model constructed using multivariate logistic regression has an AUC of 0.911 (0.870–0.951) and 0.893 (0.840–0.962), accuracy of 84.4% and 79.7%, sensitivity of 90.8% and 87.1%, and specificity of 80.5% and 79.0% in the training and validation sets, respectively.

**Conclusion:**

Machine learning models can preoperatively predict the condition of LVI effectively in patients with ESCC based on enhanced CT radiomics features. The GNB and LR models exhibit good stability and may bring a new way for the non-invasive prediction of LVI condition in ESCC patients before treatment.

## 1 Introduction

Esophageal cancer, a prevalent tumor of the digestive tract, exhibits significant variation in incidence and mortality rates across different countries (1). Esophageal adenocarcinoma (EA) has a high incidence in Western countries, while esophageal squamous cell carcinoma (ESCC) is more common in China (2). Radical esophagectomy is the main method for the treatment of esophageal cancer (3). However, the incidence of local recurrence or distant metastasis is more than 50% in patients who underwent radical esophagectomy within 3 years (4). The main routes of recurrence and metastasis of esophageal cancer are through blood vessels and lymphatic vessels. Lymphovascular invasion (LVI) performs a vital role in tumor cell dissemination and lymphatic metastasis and is associated with an increased risk of micrometastasis (5, 6). LVI is an important risk factor for poor prognosis of esophageal cancer patients, with poor overall survival and progression-free survival (7, 8). Since LVI-positive patients have a higher recurrence rate, LVI-positive patients need adjuvant therapy and close monitoring before operation. Early recognition of high-risk recurrence patients is essential for the development of personalized ESCC treatment. Some studies (9, 10) have used the image features of CT to predict the condition of LVI of esophageal cancer before surgery, but the accuracy of these morphological characteristics in predicting LVI is still not ideal, and it is hard to accurately reveal the tumor structural heterogeneity. Recently, radiomics and machine learning algorithms have been rapidly developed and widely used, which is an important development direction of tumor translational medicine in the future.

Previous studies have shown that radiomics features in predicting the condition of LVI in many solid tumors (11–13) have potential clinical value. At present, there are few studies on whether we can use radiomics features to predict the condition and prognosis of LVI in esophageal cancer patients, which is of vital importance for clinical medicine strategy making. Therefore, this study aims to explore the value of a machine learning model based on enhanced CT radiomics features in the prediction of LVI condition in ESCC patients preoperatively, so as to guide the development of clinical personalized treatment strategy and improve patient prognosis.

## 2 Materials and methods

### 2.1 Data acquisition

Data from 258 ESCC patients who were confirmed by pathology with radical esophagectomy from June 2017 to December 2019 were retrospectively collected. The selection criteria included the following: a) tumor was resected with radical resection and postoperative pathology confirmed it to be ESCC, b) clinical and imaging data were complete, and c) immunohistochemistry (IHC) and staining with hematoxylin–eosin (HE) were performed with complete pathological results. The exclusion criteria included the following: a) no basic clinical data or CT-enhanced images before surgery, b) other pathological subtypes of esophageal cancer, c) esophageal lesions were small and could not accurately delineate the area of interest, and d) any other treatment was performed before surgery. Finally, 258 patients were enrolled in this study, consisting of 161 men and 97 women ages 47 to 81 with a median age of 64.5 years, and 146 were LVI-positive and 112 were LVI-negative. All patients were divided into a training set (182 cases) and a validation (76 cases) set at a ratio of 7:3 ratio. Clinical baseline data were collected, including gender, age, and smoking and drinking history.

### 2.2 CT image acquisition and analysis

All 258 patients were scanned using the Siemens Somatom Definition CT scanner (Munich, Germany). The scanned area covers the entire esophagus from the thoracic entrance to the floor of the diaphragm, and the patient lies on his back and holds his breath once to complete the scan. The scanning parameters were as follows: slice thickness, 5 mm; slice spacing, 5 mm; pitch, 1.5:1; rotation time, 0.5 s; scanning field, 350 × 350 mm; matrix, 512 × 512; tube voltage, 120 kVp; and tube current, 130 mAS. A high-pressure syringe was used to inject 1.5 ml/kg of contrast agent, and an enhanced CT scan for 25–30 s (arterial stage) was carried out after injecting the contrast agent. We send all images to the picture archiving and communication system (PACS) workstation. Two radiologists performed image analysis without knowing clinical data, pathology, or LVI status. Normal dilated esophageal walls are approximately 3 mm thick, whereas in esophageal cancer, the wall is significantly thickened or mass-like, and a thickened wall of more than 5 mm is considered abnormal (14). The following CT features were then observed and recorded: a) tumor location: the tumor location is defined by the location of the tumor center in the esophagus; b) tumor size: the tumor thickness is measured based on enhanced CT (if the lumen is completely occluded, measurement of the tumor’s maximum diameter is done at one-half of its maximum transverse section; when there is a gap between the tumor and esophageal cavity, the tumor’s maximum diameter is measured by the margin of the thickened wall to its cavity at the maximum transverse section); c) the enhancement ratio of tumors to normal walls (TNR): TNR is figured out through dividing the average tumor CT value by the average CT value of a normal esophageal wall to measure the contrast enhancement difference between the tumor and the normal esophageal wall. We placed the region of interest (ROI) in an area with the greatest cross-section of arterial phase enhancement, avoiding vascular structure, ulceration, and necrosis; d) clinical T staging refers to the clinical T staging standard put forward by Griffin et al. (15). Clinical N staging was determined according to different regions’ metastatic lymph node counts, and metastatic lymph nodes were determined according to the ratio between the shortest diameter and the axis of enlarged lymph nodes (16, 17). Clinical AJCC staging refers to the AJCC/UICC 8th edition cancer staging criteria (14).

### 2.3 ROI delineation and radiomics features extraction

We uploaded all CT images to the open-source software ITK-SNAP (www.itksnap.org). Two experienced radiologists manually delineate the ROI along the margin of the tumor layer by layer for tumor segmentation. All lesion areas should be included in the three-dimensional ROI but should avoid fatty tissue, lymph nodes, blood vessels, luminal gas, and fluid around the lesion. Image preprocessing and radiomics feature extraction use the open-source software FAE based on PyRadiomics (version 0.5.2; https//github.com/salan668/FAE) (18). Esophageal peristalsis and large vessel pulsation are two interference factors that may be encountered in the process of radiomics feature extraction of esophageal cancer, which may have an impact on the stability of feature extraction. Esophageal peristalsis may cause motion blur and deformation of the images, which may interfere with the extracted radiomics features. Therefore, it may be necessary to adopt appropriate motion correction algorithms or image reconstruction techniques when extracting features in order to reduce the influence of esophageal peristalsis. Large vessel pulsations often cause vibration or pulsation artifacts of the images, which may interfere with feature extraction. This problem can be addressed by using filtering algorithms or motion correction techniques to reduce or eliminate interference from large vessel pulsations. Two hundred sixteen first-order features, 1,086 texture features, and 14 shape features were extracted. Texture features include the gray-level dependence matrix (GLDM), gray-level co-occurrence matrix (GLCM), gray-level run length matrix (GLRLM), gray-level size zone matrix (GLSZM), and neighboring gray tone difference matrix (NGTDM). Based on the training set data, all feature parameters were normalized with *Z*-score. To ensure intraobserver stability of radiomics features, radiologist 1 randomly selected 30 patients with CT images for segmenting ROI independently and extracting features in 1 week. In order to ensure the interobserver stability of the radiomics feature, radiologist 2 segmented ROI independently and extracted features on CT images of 30 randomly selected patients. The repeatability of feature extraction was assessed by intraobserver and interobserver correlation coefficients (ICCs).

### 2.4 Radiomics features selection and model construction

A variety of algorithms were used to reduce the dimensionality of high-dimensional data: 1) selected features were those with high stability in both intraobserver and interobserver consistency tests for analysis (ICCs > 0.90); 2) in the combination of MRMR, the first 20 image omics feature parameters with feature score or importance ranking were selected; and 3) the label of the dataset was constructed by the LASSO regression model, and finally, the radiomics score of each case was obtained.

In machine learning model construction, five machine learning algorithms—1) SVM, 2) KNN, 3) LR, 4) GNB, and 5) MLP—were used to model the optimal feature subset and statistically significant clinical features. We used ROC curves to evaluate the predictive power of the machine learning model for ESCC with LVI, and the AUC, accuracy, sensitivity, specificity, F1 score, and Kappa value were calculated.

### 2.5 Statistical analysis

SPSS 22.0 and R statistical software version 3.6.3 were used for all statistical analysis. Quantitative data consistent with normal distribution were represented by $\bar{x}$ ± *s*. Qualitative data were represented by frequency. In the clinical data analysis, the independent sample t-test was used for quantitative data with normal distribution, non-conforming quantitative data were analyzed by the Mann–Whitney *U* test, and categorical variables were analyzed by the *χ* ^2^ test. Radiomics features were screened by LASSO regression 10-fold cross-validation in the “glmnet” package of R software. The software packages (including “class,” “kernlab,” “e1071,” “stats,” and “nnet”) were used to implement machine learning algorithms such as KNN, SVM, GNB, LR, and MLP. The ROC curves of all machine learning models were analyzed, and AUC, accuracy, sensitivity, specificity, and F1 scores were calculated. We drew calibration curves for different combination models based on their goodness of fit. Calibration curve reliability was assessed by Hosmer–Lemeshow. The clinical effectiveness of the three models was quantified using decision curve analysis (DCA) under different threshold probabilities. The statistical significance levels were all bilateral, and statistical significance was indicated by *P <*0.05.

## 3 Results

### 3.1 Clinical data

Statistically significant differences were found in tumor thickness, TNR, and clinical N stage among 258 ESCC patients (*P* < 0.05), but there were no significant differences in age, gender, smoking history, drinking history, tumor location, clinical T stage, and clinical AJCC stage (*P* > 0.05). Multivariate logistic regression analysis revealed that tumor thickness (OR = 1.189, 95% CI 1.060–1.351, *P* = 0.005), TNR (OR = 2.966, 95% CI 1.174–7.894, *P* = 0.024), and clinical N stage (OR =5.828, 95%CI 1.752-20.811, *P* = 0.005) were the independent predictors of the occurrence of LVI (**Table 1**).

**Table 1:** Analysis of clinical features based on univariate and multivariate logistic regression.

	Univariate analysis		Multivariate analysis	
	OR value (95% CI)	*P-*value	OR value (95% CI)	*P-*value
Age	1.007 (0.968, 1.049)	0.718	–	–
Gender		0.745	–	–
Female	Ref		–	–
Male	1.099 (0.584, 2.091)	0.771		
Smoking history		0.874		
No	Ref		–	–
Yes	1.125 (0.609, 2.096)	0.708	–	–
Drinking history		0.984		
No	Ref		–	–
Yes	1.066 (0.583, 1.959)	0.836	–	–
Tumor location		0.088		
Upper	Ref		–	–
Middle	0.520 (0.217, 1.224)	0.136	–	–
Lower		0.123	–	–
Tumor thickness	1.276 (1.170, 1.406)	<0.001*	1.189 (1.060, 1.351)	0.005
TNR	6.622 (2.929, 16.139)	<0.001*	2.966 (1.174, 7.894)	0.024
Clinical T stage	NA	NA	–	–
Clinical N stage		<0.001*		
N0	Ref	<0.001	Ref	<0.001
N1	4.031 (2.044, 8.132)	0.003	5.828 (1.752, 20.811)	0.005
N2	6.613 (2.050, 25.559)	0.987	7.832 (1.202, 61.733)	0.039
N3	NA		1.536 (0, NA)	1
Clinical AJCC stage	NA	NA	–	–

TNR, tumor-to-normal wall enhancement ratio; AJCC, American Joint Committee on Cancer; OR, odd ratio; CI, confidence interval; Ref, reference; NA, not available.

*P-value <0.05.

### 3.2 Radiomics feature selection and signature construction

We extracted 1,316 features from the preoperative enhanced CT images of 258 ESCC patients. First, we selected the features with high stability in both intraobserver and interobserver consistency tests for analysis (ICCs >0.90). Then, by integrating the radiomics features of the top 20 MRMR scores, LASSO was used for further dimensionality reduction. A feature subset with the greatest predictive ability was selected, and the associated coefficients were calculated. Finally, 14 important radiomics features were selected, and in order to calculate the Rad-score, the weighted coefficients of the selected features were summed. In both training and validation sets, the Rad-score of the LVI-positive group was significantly greater than that of the LVI-negative group, with a statistical difference (*P* < 0.05). The diagnostic efficacy of the Rad-score for predicting LVI condition was 0.858 (95% CI 0.798–0.905) in the training set and 0.876 (95% CI 0.780–0.940) in the validation set.

### 3.3 Diagnostic efficacy of different machine learning models for predicting the LVI condition of ESCC

**Figure 1:**
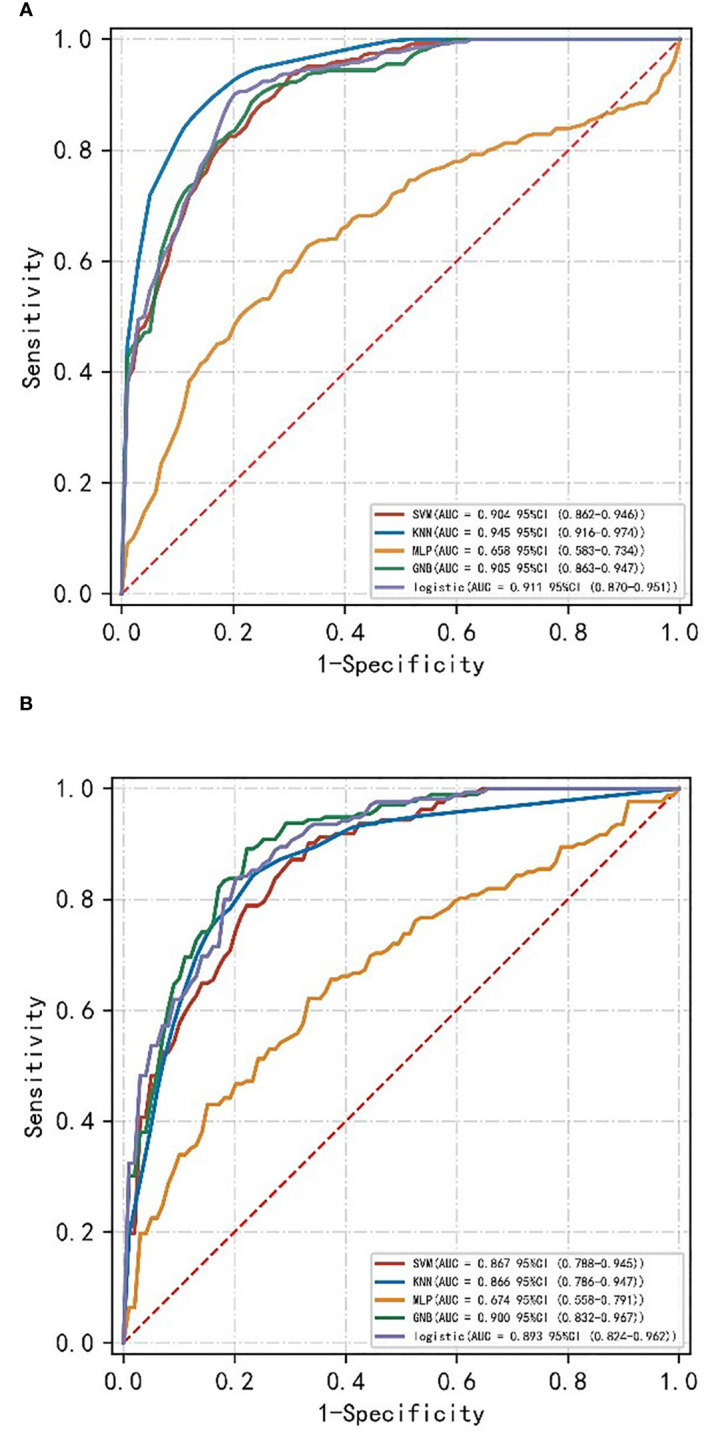
ROC curves of different machine learning models of the training set **(A)** and validation set **(B)**.

**Figure 2:**
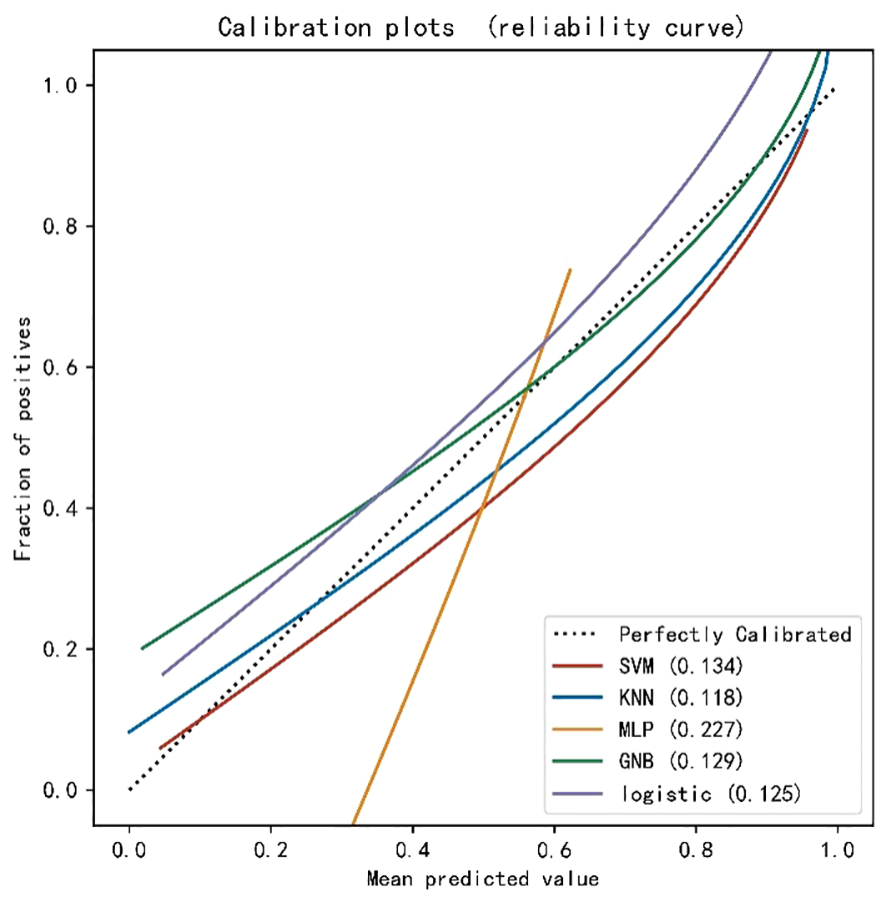
Calibration curves of the five machine learning models in the test set.

**Figure 3:**
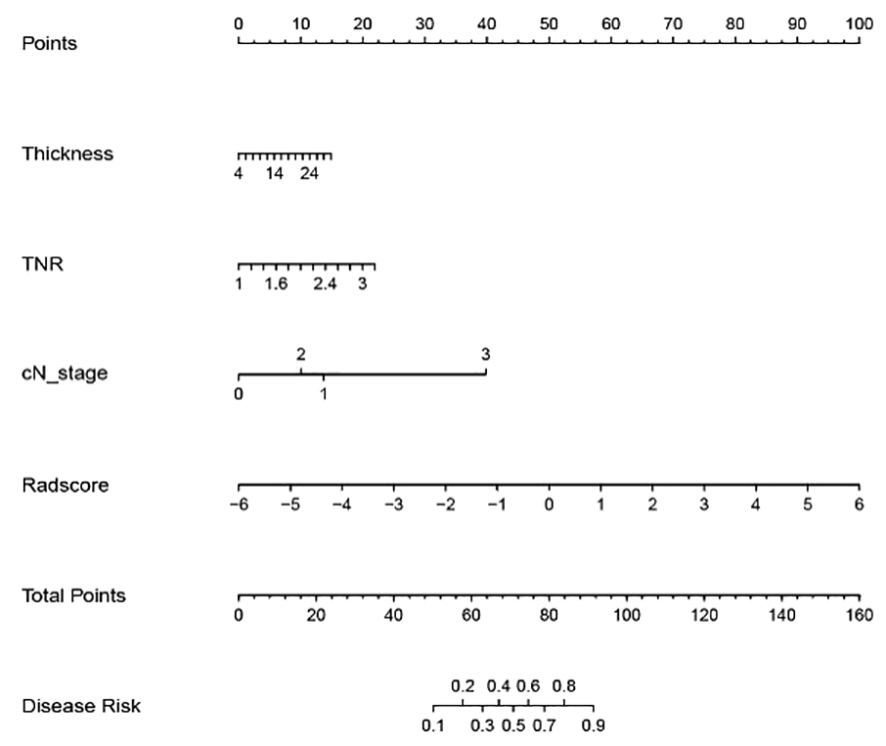
Nomogram of the multiscale combination model to predict ESCC with LVI.

A machine learning model was constructed by the combination of radiomics label constructed from the 14 best radiomics features based on enhanced CT images and 3 independent risk factors for LVI in ESCC patients of clinical characteristics. The diagnostic efficiency and the ROC curves of five machine learning models in the prediction of LVI condition of ESCC are shown in **Table 2**; **Figure 1**. Among all the models, SVM, KNN, LR, and GNB have better AUC values with high sensitivity, specificity, and accuracy in the test set, showing that these four machine learning models have higher prediction efficiency, while the MLP model has poor performance. The AUC values of the KNN and SVM models were 0.945 and 0.905 in the training set but decreased to 0.866 and 0.867 in the validation set, which showed an obvious overfitting phenomenon. In the training and test sets, the AUC values of the GNB and LR models were 0.905, 0.900, 0.911, and 0.893, respectively. The model performance was relatively stable, and there was no overfitting, but with good fitting and prediction ability (**Figure 2**). Based on the above machine learning model results, a multiscale combination model (nomogram) was constructed by multivariate logistic regression, including tumor thickness, TNR, clinical N stage, and radiomics score (**Figure 3**). As the actual curve approaches the reference line, there was a good agreement between the model predictions and the pathological findings.

**Table 2:** Diagnostic efficacy of different machine learning models to predict the ESCC with LVI.

Model	Training set				Validation set			
	AUC (95% CI)	Accuracy (%)	Sensitivity (%)	Specificity (%)	AUC (95% CI)	Accuracy (%)	Sensitivity (%)	Specificity (%)
SVM	0.905 (0.862–0.94)	81.7	86.7	78.6	0.867 (0.788–0.945)	75.1	88.8	72.2
KNN	0.945 (0.916–0.97)	84.4	89.1	85.1	0.866 (0.786–0.947)	83.5	80.5	83.0
MLP	0.658 (0.583–0.73)	65.7	75.0	59.6	0.674 (0.558–0.791)	61.8	59.3	75.2
GNB	0.905 (0.863–0.94)	83.1	87.7	80.5	0.900 (0.832–0.967)	81.8	91.6	78.8
LR	0.911 (0.870–0.95)	84.4	90.8	80.5	0.893 (0.824–0.962)	79.7	87.1	79.0

AUC, area under the curve; CI, confidence interval; SVM, support vector machine; KNN, k-nearest neighbor; MLP, multilayer perceptron; GNB, Gauss naive Bayes; LR, logistic regression.

**Figure 4:**
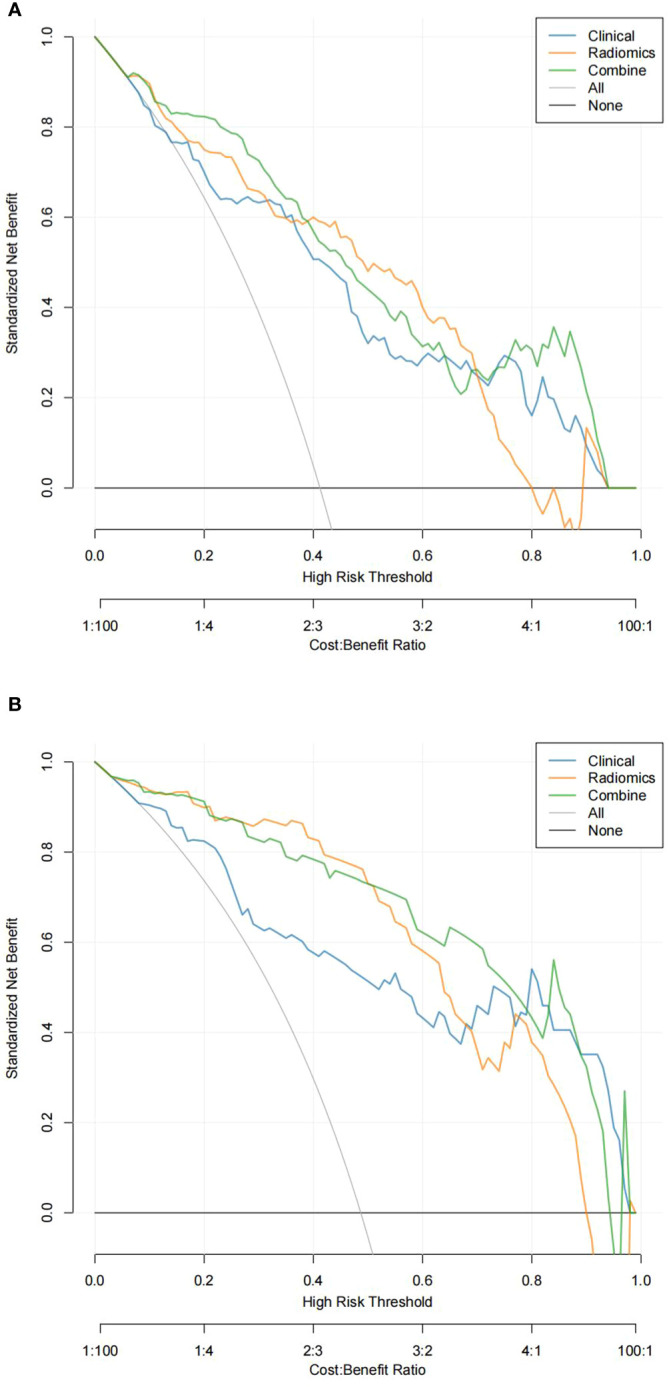
Decision curve analysis (DCA) of the training set **(A)** and validation set **(B)**. In DCA, the abscissa represents the range of risks that may be predicted, and the ordinate represents the net benefits.

In the training and validation sets, the nomogram model predicted that the AUC of LVI was 0.911 (0.870–0.951) and 0.893 (0.840–0.962), the accuracy was 84.4% and 79.7%, the sensitivity was 90.8% and 87.1%, and the specificity was 80.5% and 79.0%, respectively. Decision curve analysis showed that in the training set and validation set, the nomogram model integrating clinical factors, CT image features, and radiomics features had the greatest benefit and had a higher overall benefit than the single clinical model and radiomics model. The use of the nomogram model to predict LVI would be more beneficial than the “treatment-all” or “treatment-none” scheme (**Figure 4**).

## 4 Discussion

LVI is an independent prognostic factor for patients with ESCC (6, 19). Currently, although LVI was not included in the AJCC/UICC guidelines of the TNM staging system, as a prognostic indicator for esophageal carcinoma, the prediction of LVI condition preoperatively is of vital importance for implementing an aggressive treatment program in ESCC patients. Clinically, for esophageal cancer patients with suspected LVI-positive, more active therapy is needed, including a broader scope of surgery or adjuvant therapy before surgery. At present, how to predict the occurrence of LVI non-invasively and accurately before surgery is still quite difficult. Enhanced CT is a routine examination for esophageal cancer patients, which has important value in differential diagnosis, preoperative evaluation, efficacy evaluation, and prognosis prediction.

In this study, in order to construct a radiomics label, we used LASSO to reduce the regression coefficient and then checked the correlation between predictive factors and results. This method is superior to the method of selecting predictors based on the strength of the univariate association between the predictors and the results, and it can also combine the selected features into the radiomics label (20). In our study, we screened out crucial radiomics features from 1,316 candidate features and finally selected 14 radiomics features that can predict the condition of LVI, in which the wavelet filter provides more information (*n* = 7). These results indicate that the wavelet filter provides the best radiomics information on tumor heterogeneity and is the best available option, in accordance with the results of other radiomics studies (21). Among the selected radiomics features in this study, GLSZM (*n* = 7), NGTDM (*n* = 1), GLRLM (*n* = 3), and GLCM (*n* = 1) are high-order texture features, which can accurately reflect tumor heterogeneity. Based on the final selected radiomics features, we construct the radiomics label, which has a high degree of stability and low level of redundancy and maintains a stable correlation with LVI. On the basis of the radiomics label, we construct this radiomics prediction model. The results revealed that this radiomics model showed good predictive performance in both training and validation sets, with AUC values of 0.858 (95% CI 0.798–0.905) and 0.876 (95% CI 0.780–0.940), indicating that the prediction accuracy and stability of this model are relatively high, which is superior to the above clinical model based on clinical factors and semantic features of CT images and consistent with the research results of Li et al. (22).

Machine learning is a field of artificial intelligence, which can be seen as an automated pattern recognition and prediction technology; through the learning of data, machine learning algorithms are able to analyze the rules of the data and make reasoning, decisions, or predictions based on it. At present, there have been some research studies on the use of the machine learning algorithm to predict the condition of LVI (23–26). Liu et al. (27) used arterial phase CT image features and clinical factors to build a deep learning and SVM model to predict the presence of the invasion by microvascular tissue of hepatocellular carcinoma (HCC). The results show that the deep learning model has the best effect, and its AUC value is 0.845. However, the deep learning model needs plenty of sample data to exert its prediction ability, and overfitting is easy to occur under small sample data, so it is difficult to obtain the optimal parameter values. Therefore, it is necessary to consider the limit of the sample size in practical applications. In view of insufficient data, we can improve the prediction performance of the model by the method of data enhancement and transfer learning. In our study, 14 radiomics features were screened out to construct radiomic labels using enhanced CT images of the arterial stage of esophageal cancer, and 3 clinical factors and CT image features screened out through multifactor logistic regression were combined into this model for training and validation, demonstrating the rationality of the optimal feature subset selection in this study. Furthermore, five machine learning models (i.e., SVM, KNN, LR, GNB, and MLP) were constructed. Among them, SVM, KNN, LR, and GNB had better AUC values and high sensitivity, specificity, and accuracy in the test set, showing that these four machine learning models all have higher prediction power, while the MLP model had poor performance. The feasibility of using machine learning model training in the study is suggested, which can provide a reference for the prediction of the LVI condition of ESCC before operation. In the validation set, from the perspective of model differentiation index and AUC value, the GNB model has the best effect and high accuracy, followed by the LR model, the SVM model, and the KNN model. In the training set, the AUC values of the KNN and SVM models were 0.945 and 0.905 but decreased to 0.866 and 0.867 in the validation set, which indicated an obvious overfitting phenomenon. The AUC values of the GNB and LR models in the training and test groups were 0.905, 0.900, 0.911, and 0.893, respectively. The model performance was relatively stable and had good fitting and prediction ability. Therefore, we concluded that the GNB and LR models could be used as good machine learning prediction models to predict the condition of LVI in ESCC before surgery. To further improve its predictive ability, we constructed and validated a multiscale combination model on the basis of enhanced CT radiomics features and CT image semantic features to predict LVI condition in ESCC. The results showed that the AUC of the training set was 0.911, with 90.8% sensitivity, 80.5% specificity, and 84.4% accuracy. The AUC of the validation set was 0.893, with 87.1% sensitivity, 79.0% specificity, and 79.7% accuracy. The results showed that the model predicted the results properly, and the predictive value was verified by the calibration curve, which is consistent with the pathological findings. Chen et al. (12) used arteriovenous phase contrast-enhanced CT images to build a radiomics model for predicting LVI condition in gastric cancer. Compared with our study, this model performed similarly, but our radiomics model used enhanced CT images of the single-artery phase. For esophageal cancer, venous phase contrast-enhanced CT scanning is not a routine sequence, and the single-artery phase is used more commonly in clinical practice. Our multiscale combination model can generate an individual probability for the prediction of LVI condition by integrating preoperative radiomics features, clinical risk factors, and semantic features of CT images, which can provide physicians and patients with a user-friendly scoring system for the personalized prediction of LVI risk before operation, which aligns with the present pattern of individualized healthcare.

A few limitations are present in the study. Firstly, it is a retrospective study, including only surgical patients, and there may be a certain degree of selection bias. Secondly, it is a single-center study, which means that the patient population only includes cases from one research center, with insufficient sample size and lack of external validation, so further research involving multicenter validation and a larger sample size is needed. Thirdly, only radiomics features were analyzed, and there was no integration of multiple omics such as genomics or proteomics in this study. The combined study of multiple omics can further improve the accuracy of LVI prediction of ESCC. Finally, the biological interpretability of imaging features is insufficient. How to correlate imaging features with biological characteristics and establish reliable imaging biomarkers are the next research directions.

## 5 Conclusion

LVI can better guide preoperative treatment strategies when predicted before treatment. The machine learning model on the basis of enhanced CT radiomics features can effectively predict the LVI condition of ESCC, among which the GNB and LR models have good stability, which is expected to provide a new way for non-invasive prediction of the LVI condition of ESCC before treatment. The multiscale combined model constructed by combining clinical risk factors, semantic features of CT images, and radiomics features has good predictive accuracy for the prediction of the LVI condition of ESCC before treatment. The new method will support clinical decision-making for patients with ESCC by stratifying their risk levels.

## Data availability statement

The raw data supporting the conclusions of this article will be made available by the authors, without undue reservation.

## Ethics statement

The studies involving humans were approved by the Ethics Committee of the Affiliated Huaian No.1 People’s Hospital of Nanjing Medical University (Number: KY-2022-045-01). The studies were conducted in accordance with the local legislation and institutional requirements. The ethics committee/institutional review board waived the requirement of written informed consent for participation from the participants or the participants’ legal guardians/next of kin because as a retrospective study, the patients were exempted from written informed consent.

## Author contributions

YW: Investigation, Resources, Writing – original draft. GB: Supervision, Writing – review & editing. MH: Data curation, Writing – original draft. WC: Conceptualization, Formal analysis, Methodology, Project administration, Software, Validation, Writing – original draft, Writing – review & editing.
